# Stillbirth With Group B *Streptococcus* Disease Worldwide: Systematic Review and Meta-analyses

**DOI:** 10.1093/cid/cix585

**Published:** 2017-11-06

**Authors:** Anna C Seale, Hannah Blencowe, Fiorella Bianchi-Jassir, Nicholas Embleton, Quique Bassat, Jaume Ordi, Clara Menéndez, Clare Cutland, Carmen Briner, James A Berkley, Joy E Lawn, Carol J Baker, Linda Bartlett, Michael G Gravett, Paul T Heath, Margaret Ip, Kirsty Le Doare, Craig E Rubens, Samir K Saha, Stephanie Schrag, Ajoke Sobanjo-ter Meulen, Johan Vekemans, Shabir A Madhi

**Affiliations:** 1 Maternal, Adolescent, Reproductive and Child Health Centre, London School of Hygiene & Tropical Medicine, United Kingdom; 2 College of Health and Medical Sciences, Haramaya University, Dire Dawa, Ethiopia; 3 Newcastle University, United Kingdom; 4 Centro de Investigação em Saúde de Manhiça, Maputo, Mozambique; 5 Institució Catalana de Recerca i Estudis Avançats; 6 ISGlobal, Barcelona Centre for International Health Research; 7 Department of Pathology, Hospital Clinic of Barcelona, Universitat de Barcelona; 8 Consorcio de Investigación Biomédica en Red de Epidemiología y Salud Pública, Madrid, Spain; 9 Medical Research Council, Respiratory and Meningeal Pathogens Research Unit, and Department of Science and Technology/National Research Foundation: Vaccine Preventable Diseases, University of the Witwatersrand, Faculty of Health Sciences, Johannesburg, South Africa; 10 KEMRI–Wellcome Trust Research Programme, Kilifi, Kenya; 11 Oxford University, United Kingdom; 12 Departments of Pediatrics and Molecular Virology and Microbiology, Baylor College of Medicine, Houston; 13 Department of International Health, Johns Hopkins Bloomberg School of Public Health, Baltimore, Maryland; 14 Global Alliance to Prevent Prematurity and Stillbirth, Seattle, Washington; 15 Department of Obstetrics and Gynecology, University of Washington, Seattle; 16 Vaccine Institute, Institute for Infection and Immunity, St George’s Hospital, University of London and St George’s University Hospitals NHS Foundation Trust, United Kingdom; 17 Department of Microbiology, Faculty of Medicine, Chinese University of Hong Kong; 18 Centre for International Child Health, Imperial College London, United Kingdom; 19 Department of Global Health, University of Washington, Seattle; 20 Bangladesh Institute of Child Health, Dhaka; 21 National Center for Immunization and Respiratory Diseases, Centers for Disease Control and Prevention, Atlanta, Georgia; 22 Bill & Melinda Gates Foundation, Seattle, Washington; 23 World Health Organization, Geneva, Switzerland; 24 National Institute for Communicable Diseases, National Health Laboratory Service, Johannesburg, South Africa

**Keywords:** group B *Streptococcus*, stillbirth, stillborn, mortality, estimates

## Abstract

**Background:**

There are an estimated 2.6 million stillbirths each year, many of which are due to infections, especially in low- and middle-income contexts. This paper, the eighth in a series on the burden of group B streptococcal (GBS) disease, aims to estimate the percentage of stillbirths associated with GBS disease.

**Methods:**

We conducted systematic literature reviews (PubMed/Medline, Embase, Literatura Latino-Americana e do Caribe em Ciências da Saúde, World Health Organization Library Information System, and Scopus) and sought unpublished data from investigator groups. Studies were included if they reported original data on stillbirths (predominantly ≥28 weeks’ gestation or ≥1000 g, with GBS isolated from a sterile site) as a percentage of total stillbirths. We did meta-analyses to derive pooled estimates of the percentage of GBS-associated stillbirths, regionally and worldwide for recent datasets.

**Results:**

We included 14 studies from any period, 5 with recent data (after 2000). There were no data from Asia. We estimated that 1% (95% confidence interval [CI], 0–2%) of all stillbirths in developed countries and 4% (95% CI, 2%–6%) in Africa were associated with GBS.

**Conclusions:**

GBS is likely an important cause of stillbirth, especially in Africa. However, data are limited in terms of geographic spread, with no data from Asia, and cases worldwide are probably underestimated due to incomplete case ascertainment. More data, using standardized, systematic methods, are critical, particularly from low- and middle-income contexts where the highest burden of stillbirths occurs. These data are essential to inform interventions, such as maternal GBS vaccination.

## Teaser

Group B *Streptococcus* (GBS) is associated with invasive disease in utero and stillbirth. We estimated that 1% of all stillbirths in developed countries and 4% in Africa were associated with GBS.

There have been substantial reductions in under-5 childhood deaths worldwide, driven by the Millennium Development Goals, which ended in 2015 [1]. However, the burden of stillbirths was not included in these goals and is considerable, with around 2.6 million stillbirths each year [2], similar to the number of deaths occurring during the neonatal period (2.7 million) [3]. Most stillbirths occur in low- and middle-income contexts, in sub-Saharan Africa (1.0 million) and South Asia (1.3 million).

Data on the causes of stillbirth are limited, and comparability of causes is challenging due to multiple classification systems [4]. Obstetric emergencies, including antepartum hemorrhage and maternal hypertensive disorders (preeclampsia and eclampsia), are important contributors [4]. Infection is also important, but apart from estimates for the contribution of maternal malaria, syphilis, and human immunodeficiency virus (HIV) [4], data on infectious causes of stillbirth are sparse [5].

Group B *Streptococcus* (GBS; *Streptococcus agalactiae*) maternal colonization of the genitourinary tract is common, occurring in approximately 10%–40% of women worldwide [6, 7]. Vertical transmission leads to high incidence of early onset (0–6 days of age) neonatal GBS disease (EOGBS), essentially (80%–90% of cases) manifesting within 24 hours after birth [8]. GBS has more recently been identified as an important pathogen in neonatal disease in low-income contexts, including sub-Saharan Africa and India [9, 10].

In EOGBS and GBS-associated stillbirth, infection is likely due to ascending infection in utero from the maternal genitourinary tract, starting before delivery. Whole-genome sequencing studies demonstrate that GBS isolated at birth from the skin of newborns delivered by cesarean section are identical to those colonizing the mother. Furthermore, stillbirths with GBS isolated from postmortem blood culture were genetically identical to maternal GBS colonizing isolates [11].

Understanding the contribution of GBS as a cause of stillbirth is important to design and implement preventive interventions. For EOGBS disease, 4 or more hours of intrapartum antibiotic prophylaxis, based either on maternal clinical risk factors or the presence of maternal GBS colonization from microbiological screening at 35–37 weeks’ gestation, is frequently given in high-income contexts [12, 13]. However, this strategy is unlikely to prevent GBS-associated stillbirth occurring before labor and/or health facility attendance, where antibiotics could be administered. In contrast, maternal vaccination could protect the fetus from invasive disease in utero. A trivalent GBS polysaccharide-protein conjugate vaccine was recently evaluated in phase 2 clinical trials among pregnant women [14].

**Figure 1.**
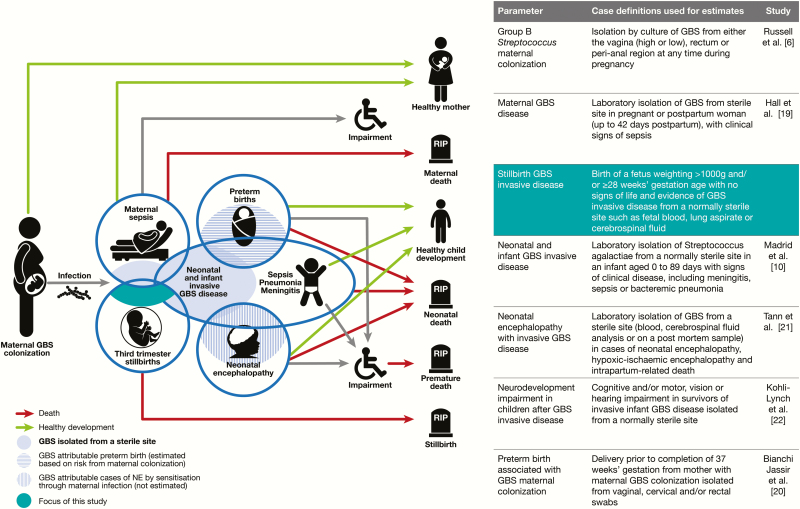
Group B *Streptococcus* (GBS)–associated stillbirth in disease schema for GBS, as described by Lawn et al [15].

We undertook a systematic review of the percentage of stillbirths associated with GBS worldwide as part of the total burden of GBS disease (Figure 1). This article is part of a supplement estimating the burden of GBS disease in pregnant women, stillbirths, and infants, which is important in terms of public health policy [15]. This supplement includes systematic reviews and meta-analyses, which form input parameters to estimates, partly through a compartmental model [16]. These are reported individually according to international guidelines for improving estimation [17, 18]: maternal colonization [6], maternal GBS disease [19], preterm birth [20], use of intrapartum antibiotic prophylaxis [12], risk of newborn disease [13], neonatal disease [10], neonatal encephalopathy [21], and impairment after neonatal disease [22]. These are used for estimates of the burden of GBS in pregnant women, stillbirths, and infants worldwide [16].

The objectives of this review were (1) to undertake comprehensive, systematic literature reviews and meta-analyses to calculate the pooled percentage of stillbirths with evidence of GBS infection regionally and worldwide; (2) to use these data for estimates of the burden of GBS in pregnancy for women, stillbirth and infants; and (3) to evaluate gaps in the data and make recommendations to improve the data on GBS-associated stillbirth.

## METHODS

This article is part of a wider study protocol entitled “Systematic estimates of the burden of GBS in pregnant women, stillbirths and infants worldwide.” It was submitted for ethical approval to the London School of Hygiene & Tropical Medicine (reference number 11966). We describe the general methods elsewhere; here we give details of methods specific to GBS-associated stillbirth.

### Definitions

We used the World Health Organization definition of stillbirth—that is, birth of a fetus with no signs of life at ≥28 weeks’ gestation or weighing ≥1000 g [23]. Where gestational data were available, this was preferred as the birthweight threshold is not equivalent to 28 weeks’ gestation [24]. Confirmed cases of GBS-associated stillbirth were based on microbiological evidence of invasive GBS disease, from a normally sterile site such as fetal blood (sampled from the umbilical cord or from the heart), lung aspirate, cerebrospinal fluid, or fetal tissues. Cases where GBS was only isolated from a potentially contaminated site (eg, placenta or amniotic fluid [other than by amniocentesis], gastric or tracheal aspirate) were not included.

### Data Searches and Inputs

We identified data through systematic review of the published literature and through development of an investigator group asking clinicians, researchers, and relevant professional institutions worldwide. For this report, we did systematic literature searches of Medline, Embase, and Literatura Latino-Americana e do Caribe em Ciências da Saúde from 15 March 2015 to 1 February 2017 to update a previous systematic review [25]. We did systematic literature searches of the World Health Organization Library Information System and Scopus on 1 February 2017. We searched databases with variants of terms related to “stillbirth/fetal mortality” and “group B *Streptococcus.*” Medical subject headings (MeSH) terms were used where possible (see Supplementary Table 1 for the full list of search terms). We did not apply language or date restrictions. We used snowball searches of article reference lists to identify additional studies [18]. Two independent investigators (A. C. S. and F. B. J.) performed the database searches, screened titles for duplicates and for eligibility, and screened abstracts to assess their suitability for inclusion, and one investigator (A. C. S.) extracted data. The data extraction from full texts was compared to a recent systematic review [25] and, for any discrepancies, a second investigator (F. B. J.) reextracted the data.

### Inclusion and Exclusion Criteria

We included studies having a defined population denominator, including all stillbirths in a facility, or occurring in a geographical location in a specified time period (Supplementary Table 2 for inclusion and exclusion criteria). We based case ascertainment on isolation of GBS identified through conventional microbiological culture. We excluded studies where only stillbirths <28 weeks’ gestation were reported (outside of the World Health Organization definition), or where cultures were only taken from potentially nonsterile or contaminated sites.

### Meta-analyses

Random-effects meta-analyses to estimate the percentage of GBS-associated stillbirth worldwide and by region were performed using the DerSimonian and Laird method [26] for recent data (from the year 2000).

### Sensitivity Analyses

Sensitivity analyses were done to assess (1) changes in time, and whether recent data (from 2000) differ in the percentage of GBS-associated stillbirth when studies reporting data from all years are included; and (2) changes by region and with time, and whether there was any difference in the proportion of stillbirth associated with GBS when studies from developed regions were categorized by year periods for median year of data collection.

## RESULTS

### Study Selection

**Figure 2.**
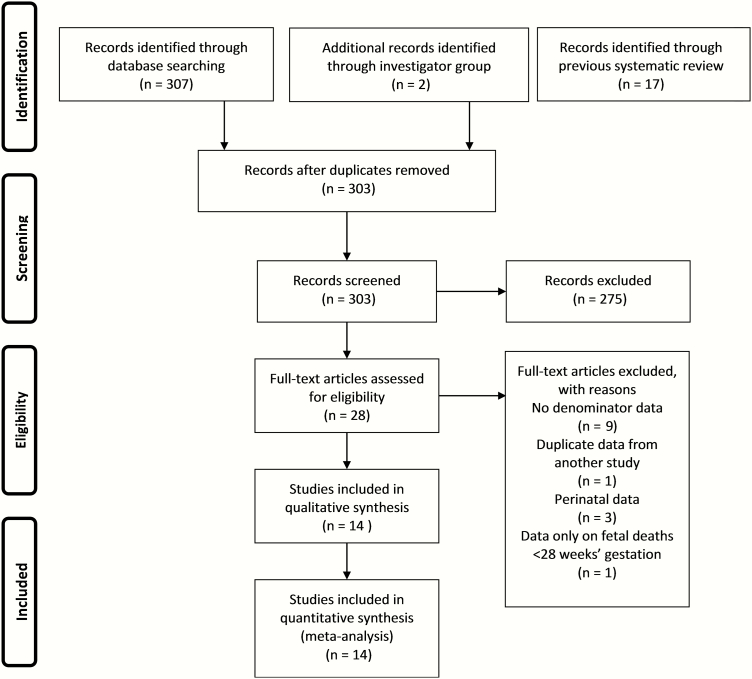
Data search and included studies on group B *Streptococcus*–associated stillbirth.

We identified 303 records through the systematic searches; 14 of these studies met the inclusion criteria. Six of the included studies (3 published articles [27–29] and 3 unpublished or updated datasets [30]) reported data collected from the year 2000 onward (recent data), whereas 8 studies reported data collected before 2000 (Figure 2 and Table 1) [31–37].

**Table 1.** Group B *Streptococcus*–Associated Stillbirth: Characteristics of Studies and Data, All Years

Study, First Author	Country	Location	Data Collection	Year, Median	Total Births	Total Stillbirths	Total Infectious Stillbirths	Total GBS-Associated Stillbirths
**Hood** [33]	United States	New Orleans	1958–1959	1958	NA	113	66	11
**Bergqvist** [39]	Sweden	Stockholm	1970–1975	1973	17638	117	24	5
**Christensen** [40]	Sweden	Lund	1979–1980	1979	130	11	2	1
**Ahlenius** [32]	Sweden	Karolinska	1987–1989	1988	10707	66	8	2
**Tolockiene** [31]	Sweden	Lund	1985–1994	1989	4130	117	32	2
**Moyo** [34]	Zimbabwe	Harare	1989–1991	1990	NA	66	43	8
**Folgosa** [35]	Mozambique	Maputo	1990–1991	1990	NA	58	41	0
**Maleckiene** [37]	Lithuania	Kaunas University	1996–1998	1997	NA	290	21	2
Embleton^a^ [30]	England	Newcastle	1981–2005	1992	906068	179	139	37
Blackwell [29]	United States	Detroit	2000–2002	2001	NA	44	NA	1
Monari [28]	Italy	Modena	2005–2011	2009	NA	109	20	4
Seale [27]	Kenya	Kilifi	2013–2014	2013	NA	149	NA	4
Madhi^a^	South Africa	Soweto	2014–2015	2014	NA	394	NA	16
Menéndez [41]	Mozambique	Maputo	2014	2014	NA	18	NA	3

Studies with all data collected before 2000 are noted in bold.

Abbreviations: GBS, group B *Streptococcus*; NA, not applicable.

^a^Includes unpublished and/or updated data from the investigator group.

### Study Characteristics

**Figure 3.**
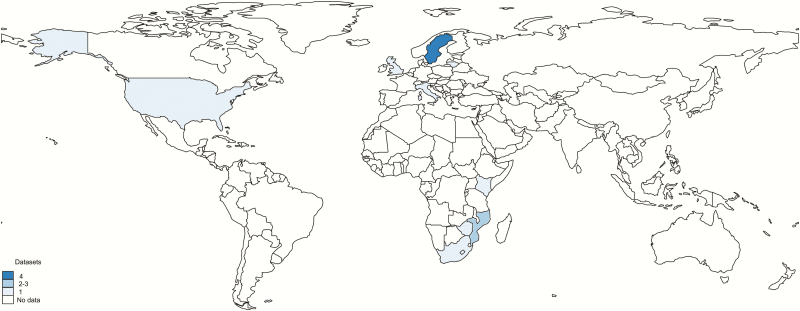
Geographic distribution of data on group B *Streptococcus* (GBS)–associated stillbirth (all years). Borders of countries/territories in map do not imply any political statement.

The characteristics of all 14 studies are summarized in Table 1. All were hospital based and included microbiological confirmation of GBS from the fetus. Most (9/14 studies) were from developed countries, with 5 of 14 studies from sub-Saharan Africa. There were no studies from Asia or South America (Figure 3).

Of the 6 studies with data collection from the year 2000, 3 were from developed countries (Italy, England, and the United States) and 3 were from sub-Saharan Africa (Kenya, South Africa, and Mozambique). Study methods differed in the details of the microbiological evidence of GBS infection. In the largest study, from England [30], cases were diagnosed based on medical case records and autopsies. In the study from Italy [34], samples were taken from heart blood and in the study from the United States, samples were amniotic fluid taken by amniocentesis [29]. In the most recent studies from Kenya, South Africa, and Mozambique, the fetus was sampled after delivery: blood from the cord or a lung aspirate in the study from Kenya [27] and cord or heart-puncture blood sampling in the study from South Africa (personal communication, S. Madhi, April 2017) and the study from Mozambique examined multiple fetal organs, with GBS detection from both conventional culture and GBS polymerase chain reaction [41].

### GBS-Associated Stillbirth

**Figure 4.**
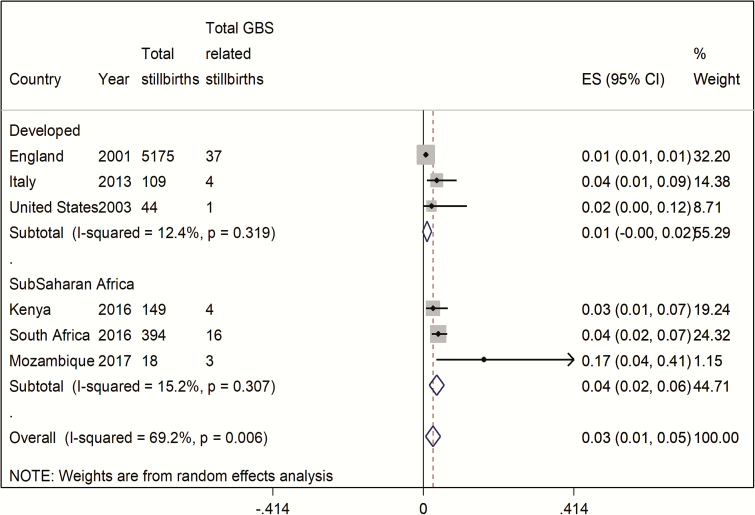
Pooled estimates (with 95% confidence interval [CI]) of proportion of group B *Streptococcus* (GBS)–associated stillbirth for regions with data since 2000, split by region (5 studies, N = 89).

The number of cases included overall was small; in studies that included data since 2000, there were 65 cases of confirmed GBS-associated stillbirth, from a total denominator of 893 stillbirths (Table 1). The percentage of GBS-associated stillbirths varied by region, being lower in developed countries with a pooled estimate of 1% (95% confidence interval [CI], 0–2%) compared to 4% (95% CI, 2%–6%) in sub-Saharan Africa (Figure 4). There was moderate heterogeneity between studies (*I*^2^ = 69%).

### Sensitivity Analyses

First, when studies reporting data from all years were included, for developed countries the pooled estimate was similar at 2% (95% CI, 1%–3%) and for sub-Saharan Africa 4% (95% CI, 1%–7%) (Supplementary Figure 1). Second, when studies from developed regions were categorized by year periods for median year of data collection, the pooled estimate was higher in the earliest studies (1961–1978) with a percentage of 6% (95% CI, 3%–10%) in stillbirths. The pooled estimate was the same in studies from 1981 to 2000 at 1% (95% CI, 0–1%) and slightly higher in 2 recent studies (2000–2017) at 3% (95% CI, 1%–3%) (Supplementary Figure 2).

## DISCUSSION

GBS is an important component of the worldwide burden of 2.6 million stillbirths, accounting for around 1% (95% CI, 0–2%) of stillbirths in developed countries and 4% (95% CI, 2%–6%) in sub-Saharan Africa. This burden and the opportunities for reduction are especially important in Africa, where the number of stillbirths is high (1.1 million), and approximately 4% are associated with GBS disease. In terms of the worldwide mortality burden of GBS, stillbirths may be far more important than neonatal deaths [16].

Estimates are limited, however, by the available data. The studies have biases in terms of access to care, samples taken, case definitions, and laboratory methods. Access to care may increase or decrease the percentage of GBS-associated stillbirth, depending on whether prenatal care reduces GBS-associated stillbirth, or if hospital delivery is more likely in mothers who notice a reduction or a cessation in fetal movements. If samples are not taken systematically, with limited numbers of sample sites (such as just fetal blood) case ascertainment will be reduced, as GBS could be detected in lung aspirate or, possibly, cerebrospinal fluid. In Mozambique, the percentage of GBS-associated stillbirth was very high (17% [95% CI, 4%–41%]), which may be due to the high number of samples taken, increasing the probability of detecting GBS [41]. While molecular methods which were used would be more sensitive, all of these cases also had GBS isolated on conventional culture, so this does not explain the difference.

An important gap is that, for much of the world, there are no data on GBS-associated stillbirth, and the wide confidence intervals in regions where there are datasets reflect the limited data from these areas However, the data gap is particularly critical in Asia. In South and Southeast Asia, >1 million of the world’s 2.6 million stillbirths occur and there is also uncertainty regarding the burden of infant GBS disease. This is reflected by lower prevalence of maternal GBS colonization, and possible differences in the virulence of GBS strains, with less serotype III, commonly associated highly invasive clonal complex 17, identified [6]. However, this is less applicable to EOGBS and GBS-associated stillbirth, where there appears from limited data to be more diverse serotypes [10, 11]. It is thus possible that there is an unrecognized burden of GBS-associated stillbirth and EOGBS disease in the first 24 hours after birth, which has not been identified with limited data on GBS disease at, or shortly after, delivery in South and Southeast Asia where, until recently, the majority of births occurred outside of health facilities.

The limited data reflect both the worldwide lack of attention to counting stillbirths [4], and to investigation of the causes of stillbirth, even in high-income contexts, where most data on this subject are historic. The lower prevalence of GBS-associated stillbirth reported in more recent data from developed regions is more likely to reflect changes in obstetric care, including increased fetal monitoring, where signs of fetal distress in utero would lead to prompt delivery and treatment. Historically this would have been less likely to be detected, as is likely now in low-income and some middle-income contexts, which may increase the proportion of GBS-associated stillbirth in these contexts, compared to EOGBS disease. This may account for more differences between contexts than intrapartum antibiotic prophylaxis, which would be likely to be given too late to reduce GBS-associated stillbirths.

The data gap for stillbirth is far greater than that for neonates, where investigations are more common, although still limited in low- and middle-income settings [10, 38]. Improving the data on GBS-associated stillbirth is critical in terms both of assessing the case for a maternal GBS vaccine and for future maternal GBS vaccine trials. Intrapartum antibiotic prophylaxis, used to reduce EOGBS disease, is unlikely to be effective for stillbirths where infection and death may occur prior to the onset of labor. Improving surveillance and research data will require standardizing sampling with consensus on the number of samples taken (and from where), as well as the use of appropriate laboratory methods, maximizing sensitivity with conventional microbiological methods and assessing the specificity of molecular methods for GBS detection in stillbirth.

## CONCLUSIONS

GBS is likely an important, potentially preventable, cause of stillbirth, especially in Africa. Improving the data across geographies, particularly South and Southeast Asia, is important, as well as establishing standard investigations and case definitions for understanding the burden of disease and for future maternal GBS vaccine trials (Table 2).

**Table 2.** Key Findings and Implications

What’s new about this? • These are the first pooled estimates of the percentage of group B *Streptococcus* (GBS)–associated stillbirth, suggesting that 4% of stillbirths in Africa may be associated with GBS, and 1% in developed regions.
What was the main finding? • GBS is an important contributor to stillbirth, particularly in Africa. GBS is likely to account for more deaths in utero, than after delivery.
How can the data be improved? • There is a critical need for more data; in terms of the geographies covered, particularly in South Asia, and in standardization of sampling strategies, laboratory methods, and case definitions.
What does it mean for policy and programs? • An effective GBS vaccine could prevent stillbirths, especially in low- and middle-income contexts.

## Supplementary Data

Supplementary materials are available at *Clinical Infectious Diseases* online. Consisting of data provided by the authors to benefit the reader, the posted materials are not copyedited and are the sole responsibility of the authors, so questions or comments should be addressed to the corresponding author.

## Supplementary Material

Supplement material

## Notes

***Author contributions.*** The concept of the estimates and the technical oversight of the series was led by J. E. L. and A. C. S. The reviews, analyses and first draft of the paper were undertaken by A. C. S. with H. B. and S. A. M. Other specific contributions were made by F. B. J., N. E., Q. B., J. O., C. M., C. C., C. B., J. E. L., and J. A. B. The GBS Estimates Expert Advisory Group (C. J. B., L. B., M. G. G., P. T. H., M. I., K. L. D., C. E. R., S. K. S., S. S., A. S.-t. M., J. V.) provided input to the conceptual process throughout, notably on the disease schema and data inputs. All the authors reviewed and input to the manuscript.

***Acknowledgments.*** We thank Claudia da Silva for administrative assistance and Alegria Perez for coordinating author signatures.

***Financial support.*** This work was supported by a grant to the London School of Hygiene & Tropical Medicine from the Bill & Melinda Gates Foundation (Grant ID: OPP1131158).

***Supplement sponsorship.*** This article appears as part of the supplement “The Burden of Group B *Streptococcus* Worldwide for Pregnant Women, Stillbirths, and Children,” sponsored by the Bill & Melinda Gates Foundation and coordinated by the London School of Hygiene & Tropical Medicine.

***Potential conflicts of interest.*** Many contributors to this supplement have received funding for their research from foundations, including the Bill & Melinda Gates Foundation, Wellcome Trust, Medical Research Council UK, Thrasher Foundation, Meningitis Research Foundation, and US National Institutes of Health. Members of the Expert Advisory Group received reimbursement for travel expenses to attend working meetings related to this series. A. S.-t. M. works for the Bill & Melinda Gates Foundation. C. J. B. has served as a member of the Presidential Advisory Committee for Seqirus Inc and of the CureVac Inc Scientific Advisory Committee, as well as undertaken consultancy work for Pfizer Inc. C. C. has received institutional compensation from Novartis for conducting GBS studies. P. T. H. has been a consultant to Novartis and Pfizer on GBS vaccines but received no funding for these activities. M. I. has undertaken sponsored research from Pfizer on pneumococcal disease in adults and from Belpharma Eumedica (Belgium) on temocillin antimicrobial susceptibility in Enterobacteriaceae. K. L. D. has received funding by the Bill & Melinda Gates Foundation to work on research on GBS serocorrelates of protection to inform vaccine trials, and travel expenses from Pfizer to attend a meeting on an investigator-led project on GBS. S. A. M. has collaborated on GBS grants funded by GlaxoSmithKline and by Pfizer and received personal fees for being member of its advisory committee; he has also collaborated on a GBS grant funded by Minervax. All other authors report no potential conflicts of interest. All authors have submitted the ICMJE Form for Disclosure of Potential Conflicts of Interest. Conflicts that the editors consider relevant to the content of the manuscript have been disclosed.

## References

[1] Liu L, Oza S, Hogan D Global, regional, and national causes of child mortality in 2000-13, with projections to inform post-2015 priorities: an updated systematic analysis. Lancet 2015; 385:430–40. 25280870 10.1016/S0140-6736(14)61698-6

[2] Colbourn T, Gilbert R An overview of the natural history of early onset group B streptococcal disease in the UK Available at: http://ovidsp.ovid.com/ovidweb.cgi?T=JS&PAGE=reference&D=emed8&NEWS=N&AN=2007109813. Accessed 1 March 2017. 10.1016/j.earlhumdev.2007.01.004 17300884

[3] Liu L, Oza S, Hogan D Global, regional, and national causes of child mortality in 2000–13, with projections to inform post-2015 priorities: an updated systematic analysis. Lancet 2015; 385:430–40. 25280870 10.1016/S0140-6736(14)61698-6

[4] Lawn JE, Blencowe H, Waiswa P Stillbirths: rates, risk factors, and acceleration towards 2030. Lancet 2016; 387:587–603. 26794078 10.1016/S0140-6736(15)00837-5

[5] Goldenberg RL, McClure EM, Saleem S, Reddy UM Infection-related stillbirths. Lancet 2010; 375:1482–90. 20223514 10.1016/S0140-6736(09)61712-8 PMC3893931

[6] Russell N, Seale AC, O’Driscoll M Maternal colonization with group B Streptococcus and serotype distribution worldwide: systematic review and meta-analyses. Clin Infect Dis 2017; 65(suppl 2):S100–11. 29117327 10.1093/cid/cix658 PMC5848259

[7] Kwatra G, Cunnington MC, Merrall E Prevalence of maternal colonisation with group B *Streptococcus*: a systematic review and meta-analysis. Lancet Infect Dis 2016; 16:1076–84. 27236858 10.1016/S1473-3099(16)30055-X

[8] Edmond KM, Kortsalioudaki C, Scott S Group B streptococcal disease in infants aged younger than 3 months: systematic review and meta-analysis. Lancet 2012; 379:547–56. 22226047 10.1016/S0140-6736(11)61651-6

[9] Sridhar S, Grace R, Nithya PJ Group B streptococcal infection in a tertiary hospital in India—1998–2010. Pediatr Infect Dis J 2014; 33:1091–2. 24776515 10.1097/INF.0000000000000377

[10] Madrid L, Seale AC, Kohli-Lynch M Infant group B streptococcal disease incidence and serotypes worldwide: systematic review and meta-analyses. Clin Infect Dis 2017; 65(suppl 2):S160–72. 29117326 10.1093/cid/cix656 PMC5850457

[11] Seale AC, Koech AC, Sheppard AE Maternal colonization with *Streptococcus* agalactiae and associated stillbirth and neonatal disease in coastal Kenya. Nat Microbiol 2016; 1:16067. 27572968 10.1038/nmicrobiol.2016.67 PMC4936517

[12] Le Doare K, O’Driscoll M, Turner K Intrapartum antibiotic chemoprophylaxis policies for the prevention of group B streptococcal disease worldwide: systematic review. Clin Infect Dis 2017; 65(suppl 2):S142–51. 10.1093/cid/cix654 PMC5850619 29117324

[13] Russell N, Seale AC, O’Sullivan C Risk of early-onset neonatal group B *Streptococcus* disease with maternal colonization worldwide: systematic review and meta-analyses. Clin Infect Dis 2017; 65(suppl 2):S152–9. 29117325 10.1093/cid/cix655 PMC5850448

[14] Madhi SA, Dangor Z, Heath PT Considerations for a phase-III trial to evaluate a group B *Streptococcus* polysaccharide-protein conjugate vaccine in pregnant women for the prevention of early- and late-onset invasive disease in young-infants. Vaccine 2013; 31(suppl 4):D52–7. 23973347 10.1016/j.vaccine.2013.02.029

[15] Lawn JE, Bianchi-Jassir F, Russell N Group B streptococcal disease worldwide for pregnant women, stillbirths and children: why, what and how to undertake estimates? Clin Infect Dis 2017; 65(suppl 2):S89–100. 29117323 10.1093/cid/cix653 PMC5850012

[16] Seale AC, Bianchi-Jassir F, Russell N Estimates of the burden of group B streptococcal disease worldwide for pregnant women, stillbirths and children. Clin Infect Dis 2017; 65(suppl 2):S200–19. 29117332 10.1093/cid/cix664 PMC5849940

[17] Stevens GA, Alkema L, Black RE; The GATHER Working Group Guidelines for accurate and transparent health estimates reporting: the GATHER statement. Lancet 2016; 388:e19–23. 27371184 10.1016/S0140-6736(16)30388-9

[18] Liberati A, Altman DG, Tetzlaff J The PRISMA statement for reporting systematic reviews and meta-analyses of studies that evaluate healthcare interventions: explanation and elaboration. BMJ 2009; 339:b2700. 19622552 10.1136/bmj.b2700 PMC2714672

[19] Hall J, Hack Adams N, Bartlett L Maternal disease with group B *Streptococcus* and serotype distribution worldwide: systematic review and meta-analyses. Clin Infect Dis 2017; 65(suppl 2):S112–24. 29117328 10.1093/cid/cix660 PMC5850000

[20] Bianchi-Jassir F, Seale AC, Kohli-Lynch M Preterm birth associated with group B *Streptococcus* maternal colonization worldwide: systematic review and meta-analyses. Clin Infect Dis 2017; 65(suppl 2):S133–42. 29117329 10.1093/cid/cix661 PMC5850429

[21] Tann CJ, Martinello K, Sadoo S Neonatal encephalopathy with group B *Streptococcus* disease worldwide: systematic review, investigator group datasets, and meta-analysis. Clin Infect Dis 2017; 65(suppl 2):S173–89. 29117330 10.1093/cid/cix662 PMC5850525

[22] Kohli-Lynch M, Russell N, Seale AC Neurodevelopmental impairment in children after group B *Streptococcus* disease worldwide: systematic review and meta-analyses. Clin Infect Dis 2017; 65(suppl 2):S190–9. 29117331 10.1093/cid/cix663 PMC5848372

[23] World Health Organization. Maternal, newborn, child and adolescent health Available at: http://www.who.int/maternal_child_adolescent/epidemiology/stillbirth/en/. Accessed 8 January 2017.

[24] Blencowe H, Cousens S, Jassir FB National, regional, and worldwide estimates of stillbirth rates in 2015, with trends from 2000: a systematic analysis. Lancet Glob Health 2016; 4:e98–108. 26795602 10.1016/S2214-109X(15)00275-2

[25] Nan C, Dangor Z, Cutland CL, Edwards MS, Madhi SA, Cunnington MC Maternal group B *Streptococcus*-related stillbirth: a systematic review. BJOG 2015; 122:1437–45. 26177561 10.1111/1471-0528.13527

[26] DerSimonian R, Laird N Meta-analysis in clinical trials. Control Clin Trials 1986; 7:177–88. 3802833 10.1016/0197-2456(86)90046-2

[27] Seale AC, Koech AC, Sheppard AE Maternal colonization with *Streptococcus* agalactiae and associated stillbirth and neonatal disease in coastal Kenya. Nat Microbiol 2016; 1:16067. 27572968 10.1038/nmicrobiol.2016.67 PMC4936517

[28] Monari F, Gabrielli L, Gargano G Fetal bacterial infections in antepartum stillbirth: a case series. Early Hum Dev 2013; 89:1049–54. 24041816 10.1016/j.earlhumdev.2013.08.010

[29] Blackwell S, Romero R, Chaiworapongsa T Maternal and fetal inflammatory responses in unexplained fetal death. J Matern Fetal Neonatal Med 2003; 14:151–7. 14694969 10.1080/jmf.14.3.151.157

[30] Embleton ND; Northern Region’s Perinatal Mortality Survey Fetal and neonatal death from maternally acquired infection. Paediatr Perinat Epidemiol 2001; 15:54–60. 11237116 10.1046/j.1365-3016.2001.00314.x

[31] Tolockiene E, Morsing E, Holst E Intrauterine infection may be a major cause of stillbirth in Sweden. Acta Obstet Gynecol Scand 2001; 80:511–8. 11380286

[32] Ahlenius I, Floberg J, Thomassen P Sixty-six cases of intrauterine fetal death. A prospective study with an extensive test protocol. Acta Obstet Gynecol Scand 1995; 74:109–17. 7900505 10.3109/00016349509008917

[33] Hood M, Janney A, Dameron G Beta hemolytic *Streptococcus* group B associated with problems of the perinatal period. Am J Obstet Gynecol 1961; 82:809–18. 13908742 10.1016/s0002-9378(16)36146-4

[34] Moyo SR, Hägerstrand I, Nyström L Stillbirths and intrauterine infection, histologic chorioamnionitis and microbiological findings. Int J Gynaecol Obstet 1996; 54:115–23. 9236308 10.1016/0020-7292(96)02705-1

[35] Folgosa E, Gonzalez C, Osman NB, Hägerstrand I, Bergström S, Ljungh A A case control study of chorioamniotic infection and histological chorioamnionitis in stillbirth. APMIS 1997; 105:329–36. 9164478 10.1111/j.1699-0463.1997.tb00578.x

[36] Bergqvist G, Holmberg G, Rydner T, Vaclavinkova V Intrauterine death due to infection with group B streptococci. Acta Obstet Gynecol Scand 1978; 57:127–8. 345729 10.3109/00016347809155890

[37] Maleckiene L, Nadisauskiene R, Stankeviciene I, Cizauskas A, Bergstrom S A case-referent study on fetal bacteremia and late fetal death of unknown etiology in Lithuania. Acta Obstet Gynecol Scand 2000; 79:1069–74. 11130090

[38] Lawn JE, Blencowe H, Oza S Every newborn: progress, priorities, and potential beyond survival. Lancet 2014; 384:189–205. 24853593 10.1016/S0140-6736(14)60496-7

[39] Bergqvist G, Holmberg G, Rydner T, Vaclavinkova V Intrauterine death due to infection with group B streptococci. Acta Obstet Gynecol Scand 1978; 57:127–8. 345729 10.3109/00016347809155890

[40] Christensen KK Infection as a predominant cause of perinatal mortality. Obstet Gynecol 1982; 59:499–508. 6281706

[41] Menendez C, Castillo P, Martínez MJ Validity of a minimally invasive autopsy for cause of death determination in stillborn babies and neonates in Mozambique: an observational study. PLoS Med 2017; 14:e1002318. 28632735 10.1371/journal.pmed.1002318 PMC5478138

