# Supplementary material for: Stillbirth With Group B Streptococcus Disease Worldwide: Systematic Review and Meta-analyses
Source: Clin Infect Dis. 2017 Nov 6;65(Suppl 2):S125–32. doi: 10.1093/cid/cix585 (PMC5850020; doi:10.1093/cid/cix585)

The burden of Group B *Streptococcus* worldwide for pregnant women, stillbirths and children

Paper 4: Stillbirth with Group B *Streptococcus* disease worldwide: systematic review and meta-analyses

Supplementary information

Contents

Systematic estimates of the burden of Group B *Streptococcus* worldwide in pregnant women, stillbirths and infants 1

Paper 4: Stillbirth associated with Group B *Streptococcus* worldwide: systematic review and meta-analyses 1

Supplementary information 1

- Supplementary Table S1: Search terms 3
- Supplementary Table S2: Inclusion and exclusion criteria 4
- Supplementary Figure S1: Meta-analysis of proportion of stillbirths associated with Group B *Streptococcus* (all years) 5
- Supplementary Figure S2: Meta-analysis of proportion of stillbirths associated with Group B *Streptococcus* by years of data collection (developed) 6

Supplementary Table S1: Search terms

Stillbirth [MeSH Term]
Fetal Death
Perinatal Death
Perinatal Mortality
AND
Streptococcus
Streptococcal
Streptococci AND (Group AND B) or agalactiae
Streptococcus agalactiae [MeSH Terms]

Supplementary Table S2: Inclusion and exclusion criteria

	**Inclusion criteria**	**Exclusion criteria**
**Population**	Number of Stillbirths (where possible, babies born ≥28 weeks' gestation or ≥1000g)	Non-representative sample
**Case definition**	Invasive GBS disease in stillbirth isolated from a sterile site, including fetal tissues/organs.	GBS only isolated from a potentially non sterile site
**Laboratory**	GBS confirmed on culture from a normally sterile site	
**Search**	No language or date restrictions	
**Article type**		Case reports

Supplementary Figure S1: Meta-analysis of proportion of stillbirths associated with Group B *Streptococcus* (all years)

Country	Year	Total stillbirths	Total GBS related stillbirths	ES (95% CI)	% Weight
Developed					
Sweden	1978	117	5	0.04 (0.01, 0.10)	6.05
Sweden	1982	11	1	0.09 (0.00, 0.41)	0.36
Sweden	1988	117	2	0.02 (0.00, 0.06)	9.19
Sweden	1995	66	2	0.03 (0.00, 0.11)	4.52
Lithuania	2000	290	2	0.01 (0.00, 0.02)	15.92
England	2001	5175	37	0.01 (0.01, 0.01)	18.54
Italy	2013	109	4	0.04 (0.01, 0.09)	6.22
USA	1961	113	11	0.10 (0.05, 0.17)	3.58
United States	2003	44	1	0.02 (0.00, 0.12)	3.49
Subtotal (I-squared = 49.5%, p = 0.045)				0.02 (0.01, 0.03)	67.87
.					
SubSaharan Africa					
Zimbabwe	1996	66	8	0.12 (0.05, 0.22)	1.89
Mozambique	1997	58	0	0.00 (0.00, 0.06)	8.67
Kenya	2016	149	4	0.03 (0.01, 0.07)	8.93
South Africa	2016	394	16	0.04 (0.02, 0.07)	12.22
Mozambique	2017	18	3	0.17 (0.04, 0.41)	0.42
Subtotal (I-squared = 63.7%, p = 0.026)				0.04 (0.01, 0.07)	32.13
.					
Overall (I-squared = 64.4%, p = 0.000)				0.03 (0.01, 0.04)	100.00

NOTE: Weights are from random effects analysis

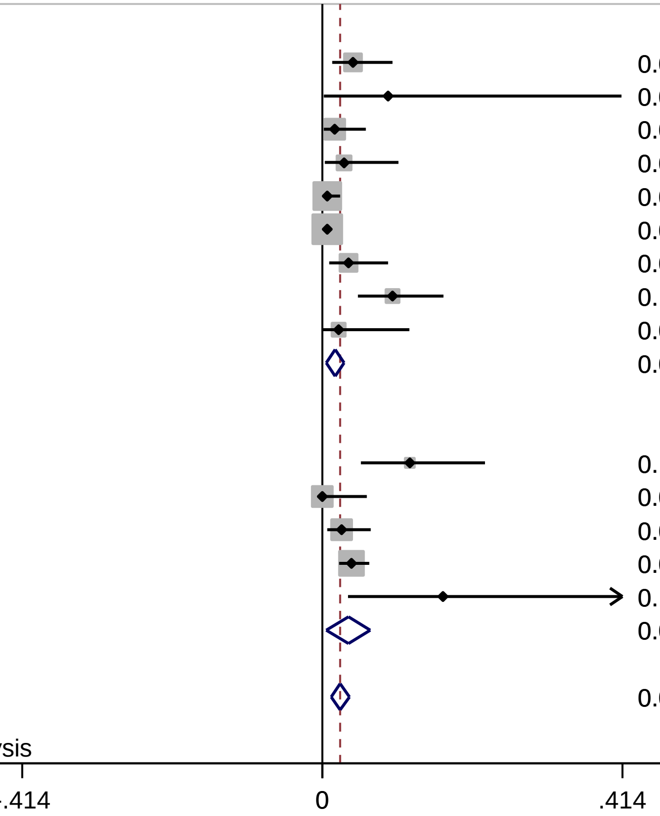

Supplementary Figure S2: Meta-analysis of proportion of stillbirths associated with Group B *Streptococcus* by years of data collection (developed)

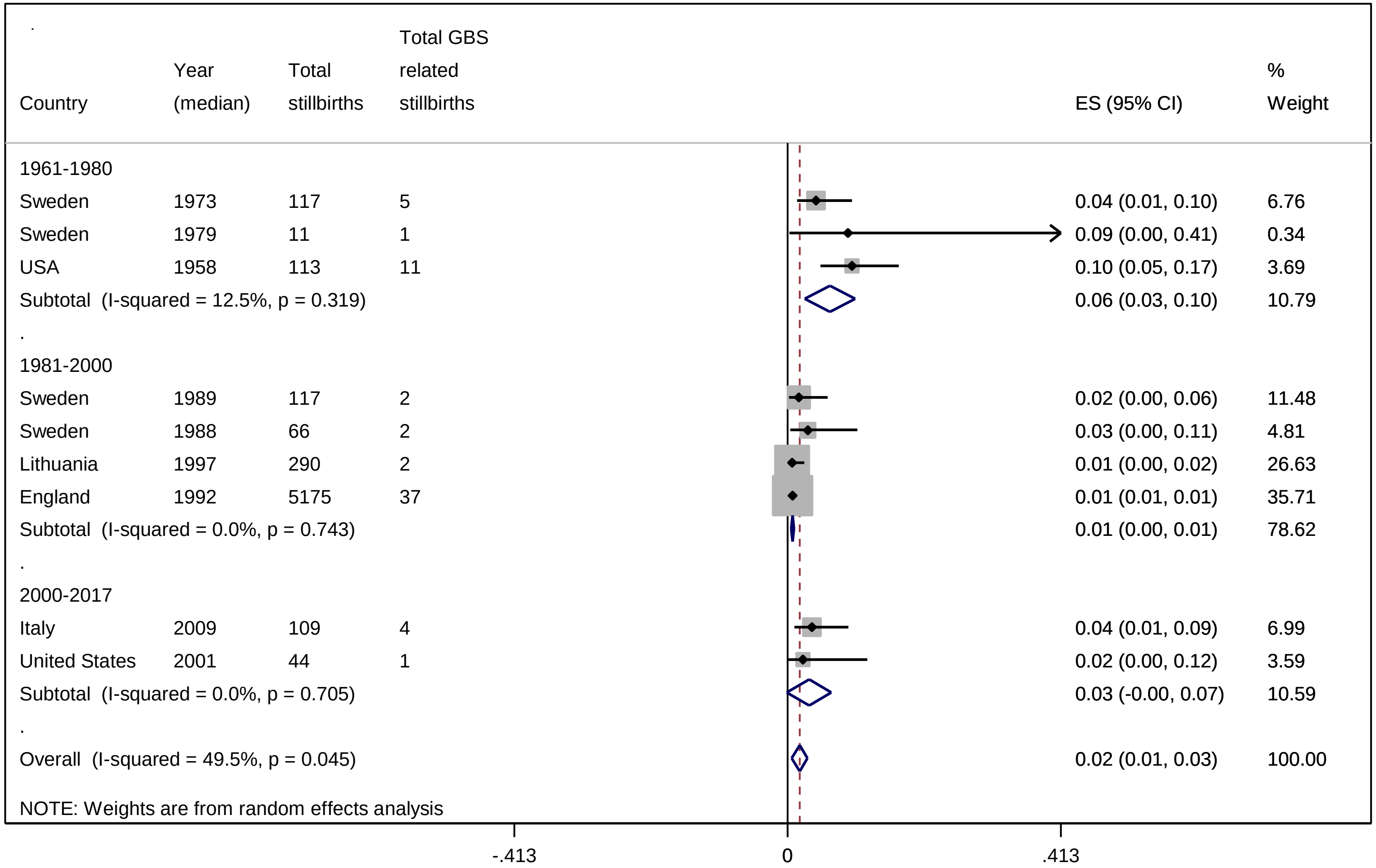

Supplement: Supplement material [file cix585_suppl_supplement-material.pdf]
